# Dissemination of OXA-23/NDM co-producing *Acinetobacter baumannii* in northern Paris hospitals: inter-hospital transmission and screening gaps

**DOI:** 10.1186/s13756-025-01694-4

**Published:** 2026-01-31

**Authors:** Marion Dutkiewicz, Claire Durand, Marie Petitjean, François Caméléna, Valentine Berti, Véronique Leflon-Guibout, Guillaume Mellon, Rishma Amarsy, Simone Nérome, Aurélie Carlier, Emmanuel Weiss, Emmanuel Dudoignon, Margaux Allain, Emilie Rondinaud, Stéphane Lo, Nathalie Grall, Noémie Mayer, Céline Ciotti, Isabelle Lolom, Signara Gueye, Luce Landraud, Frédéric Bert, Béatrice Bercot, Solèn Kernéis, Laurence Armand-Lefèvre

**Affiliations:** 1 Laboratoire de Bactériologie, AP-HP, Hôpital Bichat, 75018 Paris, France; 2 Equipe de Prévention du Risque Infectieux, AP-HP, Hôpital Bichat, 75018 Paris, France; 3 Université Paris Cité, INSERM, IAME, 75018 Paris, France; 4 Laboratoire de Bactériologie, AP-HP, Hôpital Saint Louis-Lariboisière, 75010 Paris, France; 5 Laboratoire de Microbiologie, AP-HP, Hôpital Beaujon, 92110 Clichy, France; 6 Equipe de Prévention du Risque Infectieux, AP-HP, Hôpital Saint Louis, 75010 Paris, France; 7 Equipe Opérationnelle d’Hygiène, APHP, Hôpital Lariboisière, 75010 Paris, France; 8 Equipe de Prévention du Risque Infectieux, AP-HP, Hôpital Beaujon, 92110 Clichy, France; 9 Diabétologie, AP-HP, Hôpital Bichat, 75018 Paris, France; 10 Anesthésie Réanimation, AP-HP, Hôpital Beaujon, 92110 Clichy, France; 11 Anesthésie Réanimation, AP-HP, Hôpital Saint Louis, 75010 Paris, France; 12 Laboratoire de Microbiologie Hygiène, AP-HP, Hôpital Louis Mourier, 92025 Colombes, France

**Keywords:** Carbapenem-resistant *Acinetobacter baumannii* (CRAB), OXA-23/NDM, Whole genome sequencing (WGS), Outbreak

## Abstract

Carbapenem-resistant *Acinetobacter baumannii* (CRAB) are rare in France and are usually reported in intensive care units (ICU). In 2021/2022, an unexpected increase in the incidence of CRAB isolates co-producing OXA-23 and NDM carbapenemases (OXA-23/NDM-CRAB) in several hospitals in the north of Paris prompted a common retrospective investigation. These strains were extremely resistant to both first- and second-line antibiotics, resulting in difficult-to-treat infections. We collected all cases of OXA-23/NDM-CRABs infection/colonisation between January 2020 and December 2022 in five northern Paris hospitals. Demographic and clinical data were collected for each patient. Isolates were sequenced using Illumina and representative isolates were sequenced using Nanopore. An OXA-23/NDM-CRAB was detected in 42 patients (mean age 61 years, M/F: 1.3), 58% of whom were hospitalised in a medical ward and 42% in an ICU, within three hospitals. Of these patients, 26% (11/42) were infected with CRAB, while 74% (31/42) were colonised. Two clonal strains spread over one year: ST^Ox^231^/Pas^1 in hospital 1 (n = 12) and hospital 3 (n = 13) differing by 0–16 SNPs and ST^Ox^1632^/Pas^600 in hospital 2 (n = 13) differing by 0–17 SNPs. WGS and epidemiological investigation identified the likely index patient for hospitals 1 and 3 outbreaks as a patient repatriated from hospitalisation in Cape Verde. This patient was not screened for multidrug resistant bacteria carriage during hospitalisation in hospital 1 and was detected positive 5 days after admission to the ICU in hospital 3. All outbreaks were stopped after infection control teams’ intervention. This is the first description of OXA-23/NDM-CRAB outbreaks in metropolitan France. The simultaneous dissemination of two clonal OXA-23/NDM-CRAB strains in Parisian hospitals is unusual, particularly in non-ICU settings. Medical and nursing staffs must be sensitized to the importance of screening patients returning from abroad, including for CRAB, to prevent future outbreaks.

**Supplementary Information:**

The online version contains supplementary material available at 10.1186/s13756-025-01694-4.

## Introduction

*Acinetobacter baumannii* is a Gram-negative opportunistic pathogen recognized as a significant cause of hospital-acquired infections, including ventilator-associated pneumonia, urinary tract or bloodstream infections. These infections are mostly observed in intensive care patients and are associated with high morbidity and mortality [1]. *A. baumannii* also has the ability to survive in the environment and persist on hospital surfaces and medical equipment for prolonged periods, considerably enhancing its transmission and leading to outbreaks and long-lasting endemic situations [2]. These outbreaks occur mainly in intensive care units (ICUs), where patients with severe illness and exposed to invasive devices are more likely to be colonized or infected by *A. baumannii* [3]*.* A factor contributing to mortality is the acquisition of multiple antimicrobial resistance mechanisms, resulting in resistance to last-resort antibiotics, including carbapenems [2]. Carbapenem-resistant *A. baumannii* (CRAB) were classified by the World Health Organization (WHO) as critical priority pathogens, representing a major threat to public health and underscoring the need for new antibiotics [4]. The global dissemination of CRAB is largely driven by a few dominant international clonal lineages (ICs), IC1 and IC2 being the most prevalent [5]. In CRAB, carbapenem resistance is primarily driven by the acquisition of OXA-type carbapenemases, including variants such as OXA-23-like, OXA-40-like and OXA-58-like [6]. In metropolitan France, CRAB is rarely reported, with most strains producing OXA-23 according to data from the French National Reference Center for Antimicrobial Resistance (AMR-NRC) [7]. Although NDM-producing CRAB were initially rare, their prevalence accounted for 13% of CRAB in 2017 and 32% in 2021 [7]. The co-occurrence of OXA-23 and NDM in CRAB has been increasingly documented worldwide, but these strains remain poorly described in metropolitan France. Between 2019 and 2020, hospitals in northern Paris reported an average of 1 to 7 CRAB isolates per year, corresponding to a mean incidence of 0,04 to 0,3 isolates per 1,000 admissions. During this period, only one OXA-23/NDM-CRAB isolate was identified. However, between May and October 2022, three hospitals in northern Paris simultaneously experienced a rising incidence with evidence of cross-transmission of CRAB co-producing OXA-23 and NDM carbapenemases (OXA-23/NDM-CRAB), affecting several intensive care units (ICUs) as well as medical wards.

Local epidemiological and genomic investigations were initially conducted independently by each hospital. As bacteriologists and IPC teams from these institutions meet regularly, they became aware of the concurrent emergence of OXA-23/NDM-CRAB cases and decided to compare their respective outbreaks. To explore potential links and assess the broader circulation of OXA-23/NDM-CRAB in the area, Hospital 1 proposed a retrospective review of all OXA-23/NDM CRAB cases identified between 1 January 2020 and 31 December 2022 across the five adult hospitals in Northern Paris.

## Methods

### Setting

We conducted a retrospective study from January 2020 to December 2022 in five university teaching hospitals, all part of Assistance Publique-Hôpitaux de Paris (AP-HP) and located in the north of Paris, serving a population of over 450,000 (Figure S1): Hospital 1, (890 beds, 3 ICUs), Hospital 2, (390 beds, 2 ICUs), Hospital 3 (530 beds, 3 ICUs), Hospital 4 (815 beds, 2 ICUs) and Hospital 5 (500 beds, 1 ICU).

### Infection control measures

Following national guidelines, all AP-HP hospitals have implemented screening and management policies for multidrug- and/or extensively resistant organisms (MDRO), including extended-spectrum beta-lactamase producing Enterobacterales (ESBL-E), methicillin resistant *Staphylococcus aureus* (MRSA), carbapenemase producing Enterobacterales (CPE) and vancomycin resistant enterococci (VRE)[8]. ESBL-E screening is routinely performed in ICU at admission, then weekly until discharge. CPE screening is conducted, in both medical wards and ICUs, for patients previously hospitalised abroad within the previous year, known carriers, or those with documented contact with a carrier or an infected patient. Current guidelines do not include specific recommendations for the screening and management of CRAB. However, CRAB can be detected using the same media as for ESBL-E or CPE [9, 10] and its presence is reported when incidentally identified during routine screening of ESBL-E or CPE.

In the absence of national guidelines specifically addressing CRAB, each affected hospital implemented its own IPC protocols. As the hospitals in northern Paris belong to the same hospital group and work in close collaboration, they adopted quite similar strategies. These included targeted CRAB screening (rectal and throat swabs) of all patients in the ward following the first case detection, as well as contact precautions for confirmed carriers. In medical wards, CRAB-positive patients were placed in single rooms. At Hospital 3, which is specialised in the care of burn patients and includes several dedicated units such as a burn ICU, additional systematic skin screening (inguinal fold) was also performed due to the high risk of skin colonisation in this specific population.

### Definitions

Cases were defined as patients hospitalised or consulting at one of the five hospitals between January 2020 and December 2022, with at least one clinical or screening sample showing an OXA-23/NDM-CRAB. Cases were classified as imported if the positive sample was obtained within 2 days after admission. Otherwise, they were classified as hospital-acquired. A patient was considered as infected by the attending physician and the antibiotic stewardship staff if a diagnostic sample tested positive to OXA-23/NDM-CRAB and led to treatment. All other patients were considered colonised.

### Patient data

Demographic and clinical data were collected retrospectively for each patient, including sex, age, comorbidities (diabetes mellitus, immunosuppression, cancer, haematological malignancy, solid organ transplantation, bone-marrow transplantation, cirrhosis, renal failure and chronic haemodialysis), history of travel or hospitalisation abroad, hospitalisation in France and antibiotic treatment within the past three months.

Data on hospital stay were collected, including date of admission and discharge, and the hospital department attended before, during and after CRAB detection. Antibiotic treatment for CRAB-infected patients were also recorded.

### Microbiological methods

All non-redundant OXA-23/NDM-CRABs isolated in screening or clinical samples from January 2020 to December 2022 in the five northern Paris hospitals were collected.

#### Samples

Clinical samples were processed as recommended by the French society of Microbiology [11]. CRAB screening was carried out by rectal, throat and skin swabs (Copan, Murrieta, USA) plated on CHROMID® ESBL and CHROMID® CARBA (bioMérieux, Marcy l’Etoile, France). White colonies were identified using MALDI Biotyper mass spectrometry (Bruker-Daltonics, Billerica, USA). All MDRO isolated from screening and clinical samples are routinely stored at -80 °C for 3 years in all laboratories.

#### Antimicrobial susceptibility testing

Antimicrobial susceptibility testing (ticarcillin, ticarcillin-clavulanic acid, piperacillin, piperacillin-tazobactam, ceftazidime, cefepime, imipenem, meropenem, amikacin, tobramycin, gentamicin, ciprofloxacin, trimethoprim-sulfamethoxazole and minocycline) was performed, according to CASFM/EUCAST guidelines (https://www.eucast.org/), using the disc diffusion method (Oxoid, Basingstoke, UK) on Mueller–Hinton agar (BioRad, Hercules, CA, USA). Minimum inhibitory concentrations (MICs) of colistin and cefiderocol were determined using broth microdilution (UMIC® biocentric, Bruker, Billerica, USA). MICs of tigecycline were performed using E-test strips (bioMérieux, Marcy-l'Étoile, France) and susceptibility was interpreted using CASFM/EUCAST pharmacokinetic/pharmacodynamic (PK/PD) non-species-specific breakpoints.

#### Detection of carbapenemases

All imipenem resistant *A. baumannii* were classified as CRAB. Carbapenemases were screened using a lateral flow assay, RESIST ACINETO® (Coris BioConcept, Gembloux, Belgium), detecting OXA-23, OXA-40, OXA-58, and NDM enzymes.

### Whole genome sequencing (WGS)

WGS is routinely performed in hospitals 1, 2 (same platform) and 3, using Illumina technology, on all new isolates of CPE, carbapenemase-producing *Pseudomonas aeruginosa*, and CRAB, although neither sequencing nor data analysis is conducted in real time. Available genome sequences were collected from the participating centres, and isolates that had not yet been sequenced were processed by the platform of hospitals 1 and 2 to enable comprehensive genomic comparison. DNA was extracted using EZ1 DNA TISSUE KIT (Qiagen, Hilden, Germany). Libraries were prepared with the Illumina DNA Prep kit (Illumina, San Diego, CA, United States) and sequencing was performed on MiniSeq (hospital 1 and 2) or MiSeq (hospital 3) platforms (Illumina, San Diego, CA, United States). Sequence Types (ST) were determined according Oxford [12] and Pasteur [13] schemes using MLST Finder v1.84. Resistance genes were identified using DIAMOND v0.9.22.123 and Resfinder v.2020–10-205 while virulence genes were detected using ABRIcate (https://github.com/tseemann/abricate) and VFDB [14]. Annotation was realised using PROKKA v1.14.6. Specific annotation of transposons was realised using SnapGene, TBcenter and ISFinder. Sequences were compared with Roary v3.1211 to determine the core genome and IQTree v1.6.912 was used to construct a phylogenetic tree.

Three representative strains (one per epidemic strain and hospital) were selected for long read sequencing. DNA was extracted using a NucleoMag 96 Tissue kit (Macherey Nagel, Hœrdt, France) and sequencing was performed on a MinION platform (Oxford Nanopore Technologies, Oxford, UK). Genomes were reconstructed using Illumina and Nanopore reads with Unicycler v0.4.9.

## Results

### Patients/outbreaks description

**Fig. 1:**
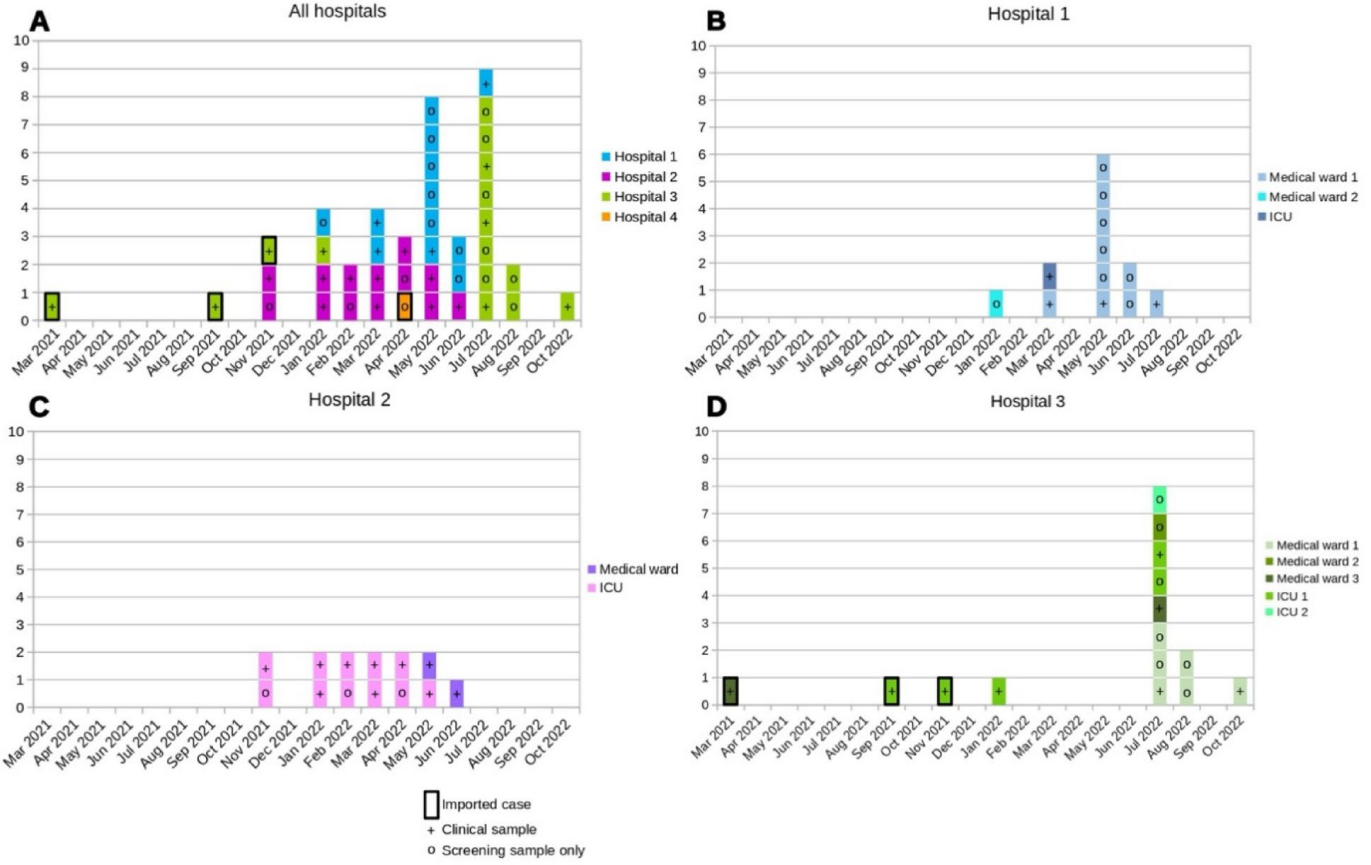
Epidemic curves of patients colonized or infected with carbapenem-resistant OXA-23/NDM-1-producing *Acinetobacter baumannii*. **A** all hospitals, **B** hospital 1, **C** hospital 2 and **D** hospital 3, from March 2021 to October 2022 (n = 42). Hospital 4 with only one patient involved is not individually represented. The patient from hospital 5 detected in February 2020 is not represented. Each box represents one patient, with the ward of hospitalisation, at the time of positive identification, indicated by different colored boxes. Boxes with bold surroundings represent imported cases. The “+” symbol indicates the positivity of at least one clinical sample (± screening sample); the “°” indicates the positivity of screening sample(s) only

On 6 May 2022, the first isolate of OXA-23/NDM-CRAB was identified in a medical department at Hospital 1, from a clinical sample (bone biopsy, diabetic foot). A second isolate was recovered from a rectal swab on May 9th, followed by four more cases detected before May 30th. This cluster prompted a local epidemiological and genomic investigation, and discussions were initiated with the IPC team of the neighbouring Hospital 2. Through these exchanges, it was discovered that a similar episode had started a few months earlier at Hospital 2 (first case on November 24th 2021) and was still ongoing, with several hygiene measures already implemented. In total, 12 CRAB cases were detected in Hospital 1 and 13 in Hospital 2. Interventions conducted by IPC team began in Decembre 2021 in Hospital 2 with the last case recorded on June 20st, 2022. In Hospital 1, interventions occurred from May to August 2022, with the last case recorded on July 12th, 2022. In June and July 2022, all OXA-23/NDM isolates from hospital 1 and 2 were sequenced. Around the same time, during the summer of 2022, Hospital 3 experienced a similar increase in OXA-23/NDM-CRAB cases and launched its own local epidemiological and genomic investigation. The IPC team implemented hygiene measures on the August 1st, 2022; a total of 15 cases were confirmed, with the last isolate identified on October 2nd, 2022. (Fig. 1).

To explore a potential low-level circulation of the strains between hospitals, we included all patients with OXA-23/NDM-CRAB detected between January 2020 and December 2022, across the five adult hospitals in northern Paris.

During this period, 42 patients, colonised or infected with an OXA-23/NDM-CRAB were identified in the five hospitals: 12 (29%) in hospital 1, 13 (31%) in hospital 2, 15 (36%) in hospital 3, and one case each in hospitals 4 and 5.

The median age of the patients was 61 years (IQR 50–77) and the sex ratio (M/F) was 1.3. The main comorbidities were diabetes mellitus (52%, 22/42) and immunosuppression (45%, 19/42); exhaustive data are presented in Table 1. Antibiotic treatment within the past 3 months was reported in 40% (17/42) of patients, including 29% (5/17) treated with carbapenems. In all, 6 patients (14%) had been hospitalised abroad, 22 (52%) in France, and 14 (33%) had not been hospitalised during the year preceding their admission. Two patients were admitted to the emergency unit and were not hospitalised (from hospitals 4 and 5). Of the remaining, 58% (23/40) were hospitalised in medical wards (mainly diabetology and dermatology) and 42% (17/40) in ICU. The median length of stay was 16 days (IQR 8–33) in medical ward and 12 days (IQR 7–25) in ICU.

**Table 1:** Demographic and clinical data from patients (n = 42)

Demographics	Total (n = 42)	Imported (n = 5)	Acquired (n = 37)
Age in years	61 (IQR 50–77)	60 (IQR 47–85)	65 (IQR 54–81)
Sex ratio (H/F)	1.3	0.7	1.5
*Comorbidities*			
Diabetes mellitus	22 (52%)	2 (40%)	20 (54%)
Immunosuppression	19 (45%)	1 (20%)	18 (49%)
Cancer	13 (31%)	1 (20%)	12 (32%)
Hematological disease	2 (5%)	0 (0%)	2 (5%)
Solid organ transplantation	10 (24%)	0 (0%)	10 (27%)
Bone-marrow transplantation	1 (2%)	0 (0%)	1 (3%)
Cirrhosis	3 (7%)	0 (0%)	3 (8%)
Chronic kidney disease	12 (29%)	0 (0%)	12 (32%)
Chronic hemodialysis	4/12 (33%)	N/A	4/12 (33%)
Within the past 3 months			
*Previous hospitalisation*			
In France	22 (52%)	0 (0%)	22 (59%)
Abroad	6 (14%)	5 (100%)	1 (3%)
Antibiotic treatment	17 (40%)	4 (80%)	13 (35%)
With carbapenem	5/17 (29%)	1/4 (25%)	4/13 (31%)
Hospitalisation			
*Hospital*			
Hospital 1	12 (29%)	0 (0%)	12 (32%)
Hospital 2	13 (31%)	0 (0%)	13 (35%)
Hospital 3	15 (36%)	3 (60%)	12 (32%)
Hospital 4	1 (2%)	1 (20%)	0 (0%)
Hospital 5	1 (2%)	1 (20%)	0 (0%)
*Type of hospitalisation*			
Emergency	2 (5%)	2 (40%)	0 (0%)
Medical ward	23 (55%)	1 (20%)	22 (59%)
ICU	17 (40%)	2 (40%)	15 (41%)
Invasive ventilation	7/17 (41%)	1/2 (50%)	6/15 (40%)
Extra-renal purification	6/17 (35%)	0/2 (0%)	6/15 (40%)
Hospital median length of stay*			
Medical ward	16 days (IQR 8–33)	2 days (IQR 2–2)	24 days (IQR 12–39)
ICU	12 days (IQR 7–25)	39 days (IQR 30–48)	14 days (IQR 8–36)
Median days to CRAB acquisition			
In medical ward (n = 22)	14 days (IQR 9–27)	*_*	14 days (IQR 9–27)
In ICU (n = 15)	9 days (4–19 days)	*_*	9 days (4–19 days)
Colonisation/Infection			
Screening samples positive only	22 (52%)	2 (40%)	20 (54%)
*Positive clinical sample*	9 (21%)	1 (20%)	8 (22%)
Colonised			
Infected	11 (26%)	2 (40%)	9 (24%)
Outcome			
In-hospital mortality	12 (29%)	0 (0%)	12 (32%)

N/A: non-applicable

^*^(i.e., from admission to discharge/death)

A total of 5 cases (12%) were initially classified as imported, all involving patients previously hospitalised in a foreign country (Egypt, Saudi Arabia, Latvia, Comoros, Cameroon) within the past three months. The remaining 37 cases (88%) were considered hospital-acquired, with a median time between admission and CRAB detection of 14 days (IQR 9–27) in medical wards and 9 days (IQR 4–19) in ICUs.

Among the 42 patients, 74% (31/42) were considered colonised: 22 (71%) were positive on screening samples only (20 rectal swabs alone and 2 both rectal and skin swabs), 4 (13%) on clinical samples only, and 5 (16%) on both clinical and screening samples. The remaining 11 patients (26%) were considered infected (4 bloodstream infections, 5 ventilator-associated pneumonias, 1 cholangitis, and 1 diabetic foot infection) and were treated (8 with colistin monotherapy, 2 with meropenem combined with either an aminoglycoside or colistin, and 1 with cefiderocol and an aminoglycoside). Infections occurred predominantly in patients hospitalised in ICUs (82%, 9/11; 6 from Hospital 2 and 3 from Hospital 3), rather than in those hospitalised in medical wards (18%, 2/11; 1 from Hospital 1 and 1 from Hospital 3). In-hospital mortality was 29% (12/42), with a median time from hospital admission to death of 16.5 days (6–153 days). Among the deceased patients, 10 were hospitalised in ICUs, and 5 had a documented CRAB infection.

### Outbreak control measures

In the three hospitals affected by the outbreaks, the infection prevention and control measures implemented were quite similar.

In ICUs, rectal swabs were systematically performed upon admission and then weekly thereafter for ESBL-E (all hospitals) and CPE (hospitals 4 and 5) screening. Since CRAB can grow on the same selective media used for ESBL-E and CPE detection, no additional rectal swabs were collected specifically for CRAB. Following the detection of an OXA-23/NDM CRAB carrier, throat swabs were also added. In Hospital 3, where the ICU specialises in the care of severe burn patients, routine skin swabs (inguinal fold) were additionally performed due to the high risk of skin colonisation in this population. In medical wards, once a second OXA-23/NDM CRAB-positive case was identified within the same unit, all patients in the ward underwent targeted screening using both rectal and throat swabs. All additional screenings were stopped after three consecutive screening rounds yielded no new positive cases in the affected unit. Across all hospitals, other potential exposure areas, such as radiology rooms or operating theatres, were not investigated for CRAB contamination. However, environmental samples were collected in Hospitals 1 and 2, including sink trap from rooms of CRAB-positive patient, surfaces in the immediate surroundings of positive patients (e.g. beds and bedside tables) and both shared and individual medical equipment (e.g. syringe drivers, stethoscopes and bandage kits).

Contact precautions were implemented for all CRAB-positive patients. Admissions and transfers were strictly limited in affected ICUs and medical wards, and spatial cohorting of positive, contact and negative patients was organized. In hospitals 1 and 2, dedicated medical and nursing teams were assigned to each cohort. In hospitals 1, 2 and 3 enhanced bio cleaning of all surfaces, furniture and shared equipment were implemented including disinfection using quaternary ammonium disinfectants, twice a day, completed with a steam cleaning after discharge of the carrier. In Hospital 1, the effectiveness of the cleaning procedures was checked by swabbing surfaces after disinfection, all of which tested negative for CRAB.

The local IPC teams of all hospitals conducted internal reviews of hygiene practices in medical wards, focusing on hand hygiene and the disinfection of surfaces and equipment. Several lapses were identified, including lack of hand hygiene compliance, excessive glove use, and inadequate disinfection of shared equipment. Refresher training sessions were subsequently provided to medical and nursing staff, emphasising best practices in hand hygiene with alcohol-based hand rubs, appropriate glove use, and effective disinfection protocols. Additionally, at Hospital 1, since the affected unit was a diabetology ward, the local IPC team also reviewed all wound care and dressing change protocols.

### Microbiology

#### Antimicrobial susceptibility testing

All isolates were resistant to currently used beta lactams, fluoroquinolones and cotrimoxazole. Regarding aminoglycosides, all strains were resistant to gentamicin and amikacin and 36% (15/42) to tobramycin. Similarly, for cyclines, all strains were resistant to tigecycline and 40% (17/42) to minocycline. Except for one isolate with a cefiderocol MIC of 8 mg/L, all MICs ranged from 2 to 4 mg/L, resulting in a resistance rate of 36% (15/42) according to CASFM/EUCAST breakpoints. No isolate was resistant to colistin.

#### Sequencing data

WGS was performed on 37 OXA-23/NDM CRAB patient isolates (five isolates not stored) and 4 environmental isolates (2 from hospital 1 and 2 from hospital 2). Analysis of the 37 sequences from patients isolates showed a core-genome of 2,506,662 bp and 2,635 genes.

#### Phylogenetic analysis

Six different sequence types (ST) were identified including four singletons and two predominant ST: ST^Ox^1632^/Pas^600 belonging to the international clone 2 (IC2) and ST^Ox^231^/Pas^1 belonging to IC1.

**Fig. 2:**
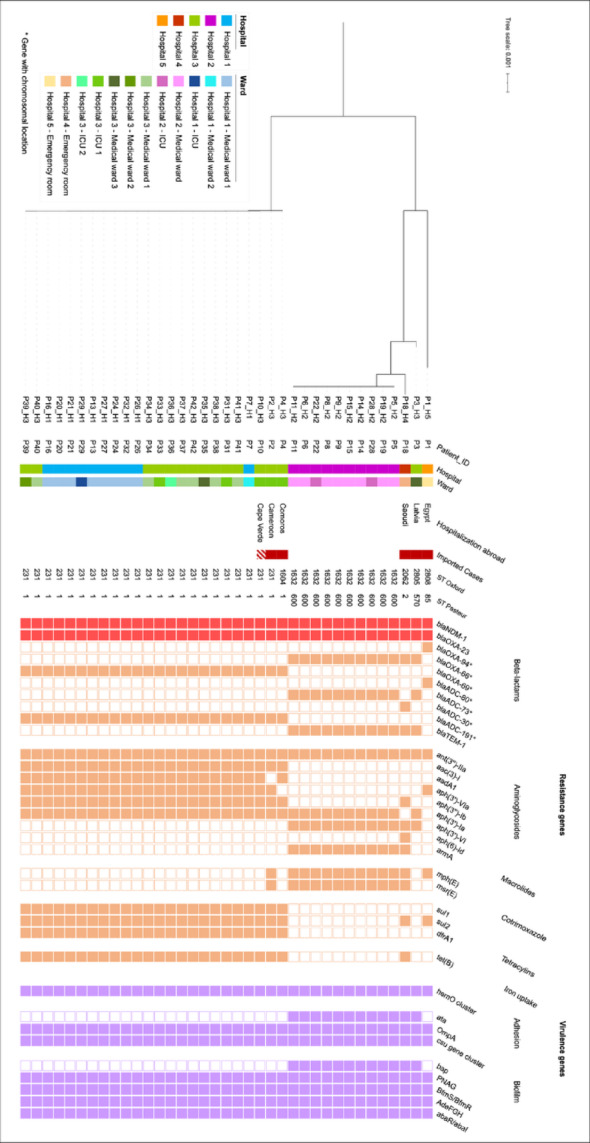
Phylogenetic tree based on the core genome of the 37 OXA-23/NDM-1-producing *Acinetobacter baumannii* isolated from patients (environmental isolates not included). Strains were collected from five adult hospitals in northern Paris between January 2020 and December 2022. The tree was constructed using a maximum-likelihood algorithm with 100 bootstrap replicates. The presence or absence of selected genes is shown as coloured or blank squares, respectively. Strip one represents the patient identification (ID); strips two and three the hospital and ward where the patient was hospitalised when the CRAB was detected; strip four the country of hospitalisation abroad during the previous 3 months; strip five the imported status of the case as confirmed (solid red) or probable (hatched red); and strips six and seven the ST according to the Oxford and Pasteur schemes, respectively. The two blocks of coloured squares indicate the presence of ARG (orange squares) and virulence genes (purple squares)

A phylogenetic tree of all patients isolates (Fig. 2) showed that five isolates were not involved in any outbreak: the four singletons and one ST^Ox^231^/Pas^1 isolate. The singletons corresponded to strains imported from abroad: Comoros (ST^Ox^1604/^Pas^1), Saudi Arabia (ST^Ox^2062/^Pas^2), Latvia (ST^Ox^2805/^Pas^570), and Egypt (ST^Ox^2808/^Pas^85). The ST^Ox^231^/Pas^1 isolate, differing from the epidemic clone by 125 SNPs, was isolated, at admission, in a patient returning from Cameroon (Fig. 2).

Two major clones were thus disseminating. The genetic distances between strains among the two STs are displayed in two SNP matrixes (Figures S2 and S3). The first clone, belonging to ST^Ox^1632^/Pas^600, included isolates differing by 0 to 17 Single Nucleotide Polymorphisms (SNPs) and accounted for 27% (10/37) of the isolates. These were mainly isolated from patients in the ICU of hospital 2 (n = 8), and patients from a medical ward in the same hospital (n = 2).

The second clone, belonging to ST^Ox^231^/Pas^1, contained isolates differing by 0 to 16 SNPs, accounted for 59% (22/37) of the isolates. These were isolated in patients from two medical wards (n = 9) and one ICU (n = 1) in hospital 1 and from three medical wards (n = 8) and two ICUs (n = 4) in hospital 3.

The sequences of the epidemic strains were compared with those available in the National Centre for Biotechnology Information (NCBI) database. The ST^Ox^231^/Pas^1 epidemic strain was closely related to a strain causing an outbreak on Réunion Island (JACOR), where the index case was a patient repatriated from the Comoros [15]. This strain differed by 100 SNPs from the epidemic clone identified in our study (Figure S4).

### Description of the two epidemic clones

#### Resistance genes

In addition to *bla*_NDM-1_ and *bla*_OXA-23_, ST^Ox^1632^/Pas^600 carried 10 additional ARGs including beta-lactamases (*bla*_ADC-73_, *bla*_OXA-66_ and *bla*_TEM-1_), and resistance genes to aminoglycosides, notably *armA*, encoding a methylase, conferring resistance to all aminoglycosides, and macrolides. ST^Ox^231^/Pas^1 carried 14 additional ARGs including beta-lactamases (*bla*_ADC-191_ and *bla*_OXA-69_), and genes conferring resistance to aminoglycosides, cotrimoxazole, tetracycline and quaternary ammonium (*qacEdelta1*) (Fig. 2).

#### Carbapenemase genes context

**Fig. 3:**
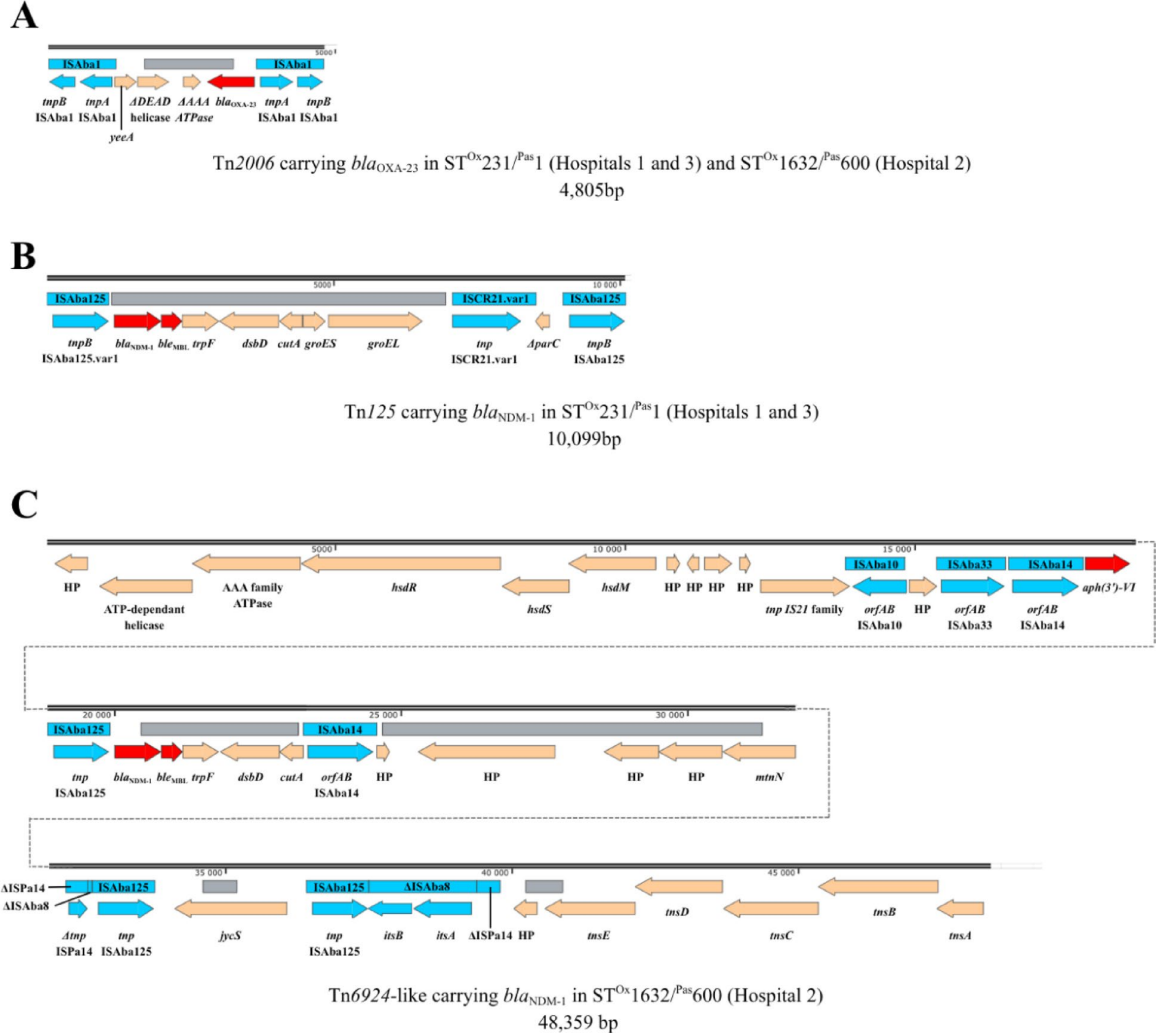
Description of **A** Tn*2006*, containing *bla*_OXA-23_ and **B** Tn*125* and **C** Tn*6924*-like containing *bla*_NDM-1_. Tn*2006* was conserved in ST^Ox^1632^/Pas^600 and ST^Ox^231/^Pas^1 isolates (4,805 bp). Tn*125* (10,099 bp) was carried by ST^Ox^231/^Pas^1 isolates and Tn*6924* (48,359 bp) by ST^Ox^1632^/Pas^600 isolates

Long-read sequencing showed that all the carbapenemase genes were located on the chromosome within different transposons. In both clones, the *bla*_OXA-23_ gene was carried by a Tn*2006* (4,805 bp), in a single copy in ST^Ox^1632^/Pas^600 and in two copies in ST^Ox^231^/Pas^1 (Fig. 3). Tn*2006* was flanked by two IS*Aba1*. In ST^Ox^1632^/Pas^600, Tn*2006* was located within a previously described resistance island, AbaR4 [16]. In ST^Ox^231/^Pas^1, the *bla*_NDM-1_ gene was carried by a Tn*125* (10,099 bp), whereas in ST^Ox^1632^/Pas^600 it was carried by a modified Tn*6924* (48,359 bp) that includes a truncated Tn*125* identical to the corresponding region of the complete Tn*125*. Tn*6924*-like also carries an *aph(3’)-VI* gene conferring a resistance to amikacin (Fig. 3).

#### Virulence genes

The epidemic strains contained multiple virulence genes, involved in iron uptake, protein secretion (type IV secretion systems), adhesion, biofilm formation and immune modulation. Most of these genes were common to both clones, except for the *bap* (biofilm-associated protein) and *ata* (adhesion) genes, only found in ST^Ox^1632^/Pas^600. Differences were also observed in immune modulation genes (Figure S4).

### WGS contribution to outbreak investigation

**Fig. 4:**
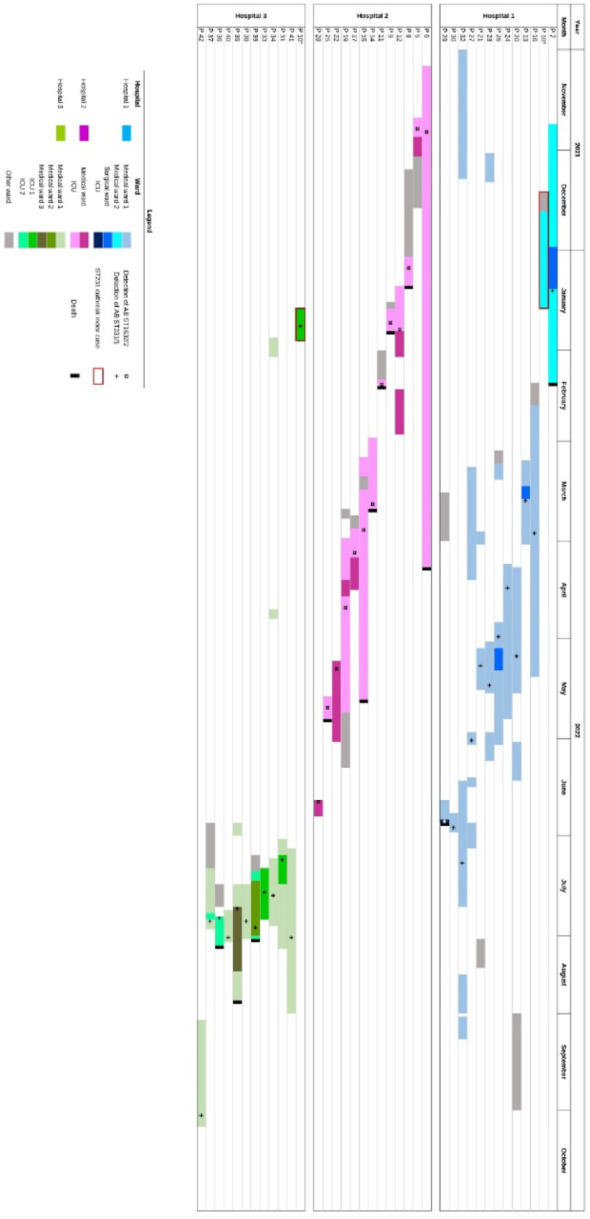
Synoptic timeline showing the patients’ trajectories and the epidemic clones disseminating among three hospitals. The coloured boxes represent the different wards in which the patients were hospitalised. The + and ¤ symbols indicate the date of detection of the epidemic strains ST^Ox^231^/Pas^1 and ST^Ox^1632^/Pas^600 respectively. The specific trajectory of patient 10 (P10), the likely index patient for the outbreaks in hospitals 1 and 3, is shown in the timelines for both hospitals

WGS clarified the likely origin of the CRAB and the epidemiological link between hospitals 1 and 3 during the outbreak caused by the strain ST^Ox^231^/Pas^1. Patient P10 was hospitalised in Cape Verde following a road traffic accident, from November 27th 2021 to December 13th 2021, before being repatriated to hospital 1 on December 14th 2021. At hospital 1, the patient was admitted to two medical wards before being transferred to the dermatology ward where he stayed from December 20th 2021 to January 19th 2022. No screening for MDROs was performed during his hospitalisation in hospital 1. On January 19th, the patient was transferred to the ICU of hospital 3, where a rectal swab taken on January 24th, five days after admission, tested positive for OXA-23/NDM-CRAB. In hospital 1, the first positive patient (P7) was identified in the dermatology ward, on January 5th, 43 days after patient P10 admission. P7 and P10 were hospitalised simultaneously in the same ward for 28 days (December 20th to January 18th). Their isolates differed by 9 SNPs. Notably, the second positive case in hospital 3 was detected on July 8th with no cases reported between January 19th and July 8th (Fig. 4).

Regarding the ST^Ox^1632/^Pas^600 outbreak, WGS confirmed the spread of a single clone but did not allow the identification of an index case.

Sequencing of environmental isolates demonstrated the presence of the epidemic strains on sinks, surfaces and medical equipment for both clones.

## Discussion

To our knowledge, this is the first report of OXA-23/NDM-CRAB outbreaks in metropolitan France, moreover occurring simultaneously across several hospitals and affecting not only ICUs but also medical wards. WGS was used to investigate this unusual increase of OXA-23/NDM-CRAB, revealing the simultaneous dissemination of two clonal strains, one circulating in hospitals 1 and 3 and the other restricted to hospital 2. Genomic and epidemiological investigations identified a likely index case for hospitals 1 and 3 and suggested that the outbreak resulted from a failure to screen at admission for MDRO carriage in a patient previously hospitalised abroad.

CRAB is one of the most serious threats related to antibiotic resistance, severely limiting therapeutic options available to treat infections, due to many strains exhibiting pan-drug resistance [2]. The global incidence of CRAB is increasing, partly driven by the widespread use of carbapenems [17]. Recently, attention has focused on CRAB harbouring multiple carbapenemases, particularly those co-producing OXA-23 and NDM [18]. These isolates have been reported predominantly in tropical and subtropical areas, including Tunisia, Egypt, Ghana, Nigeria, South Africa, India and the Indian Ocean [15, 18–23]. Our findings align with these reports, as all patients classified as imported cases returned from these regions. In France, these strains are rarely reported, and accounted for approximately 10% (around 25 isolates per year) of the CRAB analysed by the French National Reference Centre between 2018 and 2022, mostly from cases imported from the Indian Ocean and Africa [7]. CRAB is known to spread in healthcare settings, particularly in ICUs, where it is referred to as an “ICU pathogen”. Many outbreaks have been reported in Greece, Turkey, Italy and the USA, almost exclusively in ICUs and rarely in medical wards [1, 6]. A meta-analysis conducted in hospitals in southern Europe, the Mediterranean region and sub-Saharan Africa showed that the incidence of CRAB infections acquired in ICUs (40.7/1,000 patients) was 50 times higher than in medical wards (0.97/1.000 patients) [24]. While the outbreak in hospital 2 followed the typical pattern, involving patients from a single ICU, the second outbreak in hospitals 1 and 3 was unusual, primarily affecting medical wards rather than ICUs. These wards, primarily dermatology, diabetology and burn units, received patients with skin lesions likely to be CRAB colonisation sites. This observation highlights the need to systematically report CRAB when identified during CPE screening of patients recently hospitalised abroad, and to implement contact precautions for CRAB-positive patients even when admitted to medical wards.

CRAB circulation on a global scale is predominantly associated with a few high-risk international clonal lineages, especially IC2. This lineage is the most frequently described in the literature, with sequence types such as ST^Pas^2 and ST^Ox^195 commonly identified [5, 25]. IC1 is the second most prevalent, largely represented by ST^Pas^1. The strain involved in the ICU outbreak at hospital 2 belonged to ST^Ox^1632^/Pas^600 (IC2). While IC2 is frequently reported in association with healthcare outbreaks [5, 25], this specific ST has been rarely reported, with only two Egyptian strains previously described: one co-producing KPC, GIM and OXA-51 [18] and the other OXA-23 and NDM-1 [26]. By contrast, the strain involved in the outbreaks at hospitals 1 and 3 belonged to ST^Ox^231^/Pas^1 (IC1), more commonly reported and almost invariably associated with carbapenem resistance [20, 22, 27–29]. Recently, an OXA-23/NDM-CRAB outbreak in Réunion Island involved a single epidemic strain of ST^Ox^231^/Pas^1. This strain was genetically close to the one identified in hospitals 1 and 3, with 95–107 SNPs differences [15]. This outbreak affected 13 patients, including the index case transferred from Mayotte (Comoros) to Réunion Island.

The two epidemic strains identified in our study were extensively resistant to antibiotics, showing resistance to nearly all first- and second-line antibiotics, except fortunately, colistin, unlike in other outbreaks, such as that on Réunion Island [15]. MICs for cefiderocol were elevated, ranging from 2 to 8 mg/L, approaching or exceeding the EUCAST susceptibility breakpoint. Recent European guidelines conditionally recommend against the use of cefiderocol for the treatment of CRAB infections [30]. Treatments for infected patients included colistin alone, combination of meropenem with aminoglycoside or colistin, or of cefiderocol with aminoglycoside. Overall, 29% of patients died, all but two in ICU, although we did not investigate whether these deaths were directly attributable to CRAB.

In both epidemic strains, *bla*_OXA-23_ was carried by a Tn*2006* transposon. While Tn*2007* and Tn*2008* have also been associated with *bla*_OXA-23_, Tn*2006* remains the most prevalent [31]. Consistent with literature, Tn*2006 bla*_OXA-23_ was flanked by two IS*Aba1,* belonging to the IS4 family and playing an important role in the dissemination of carbapenemases genes among *A. baumannii* [32, 33]. Among *Acinetobacter* species, *bla*_NDM-1_ is almost systematically associated with Tn*125*, flanked by two IS*Aba125* [34–36]. Here, in ST^Ox^231/^Pas^1, the *bla*_NDM-1_ was classically carried by a Tn*125*. Interestingly, in ST1632^/Pas^600 the *bla*_NDM-1_ gene was located on a truncated Tn*125* integrated within a Tn*6924*-like recently described [37]. Alongside acquired β-lactamase genes, the two epidemic clones also carried distinct intrinsic *bla*_OXA-51-like_ variants. ST^Ox^231/^Pas^1 harboured *bla*_OXA-69_, commonly found in IC1 isolates and frequently reported in sub-Saharan Africa, consistent with the likely origin of the strain (Cape Verde). In contrast, ST^Ox^1632/^Pas^600 harboured *bla*_OXA-66_, typically observed in IC2 isolates and often co-occurring with *bla*_ADC-73_ [38, 39].

By strengthening comprehensive infection control measures, including patient cohorting, suspension of admissions and thorough environmental decontamination, the outbreaks were successfully stopped. The intervention of IPC teams was essential in mitigating the clinical impact of these events. To prevent future episodes, targeted education and hand hygiene training were provided to medical and nurse staff. At the time of the outbreaks, hospitals had already experienced several waves of COVID-19, and the reorganisation of services, combined with general fatigue towards infection control measures, may have contributed to procedural breaches and favoured the emergence of these outbreaks [40].

Regarding screening strategies, although these were modified during the outbreaks, notably through the addition of rectal and throat swabs, skin swabs were performed only in the ICU that routinely manages patients with severe burns. In France, skin swabs are not commonly performed, except for MRSA screening. However, evidence from the literature indicates that skin swabs may offer greater sensitivity for the detection of CRAB carriage, suggesting that current screening protocols should be reconsidered to improve early detection and strengthen infection control measures [41, 42].

The WGS investigation conducted retrospectively revealed that the increase of cases was unexpectedly due to the simultaneous circulation of two distinct clones: one confined to the ICU of hospital 2 and the other spreading to several medical units and ICUs in hospitals 1 and 3. No index case was identified for the hospital 2 outbreak, although the first two positive patients had been transferred from another French hospital that had not reported any OXA-23/NDM-CRAB. By contrast, the likely index case of the second outbreak was identified and the epidemiological link between hospitals 1 and 3 was established. This patient was transferred from a hospital in Cape Verde to hospital 1, where he was never screened for MDRO during his 28-day stay. This oversight facilitated the transmission of CRAB to another patient on the same ward, initiating the outbreak in hospital 1. The patient was later transferred to the ICU of hospital 3, where systematic screening for MDRO revealed his CRAB carriage. This patient was likely also the index case for the outbreak in hospital 3 as no other CRAB-positive patients were transferred from hospital 1 to hospital 3. However, the five-month delay before detection of the second case at hospital 3 remains unexplained, potentially attributable to the persistence of the strain in the environment or to undetected carrier patients. Nevertheless, in the absence of extensive screening, the possibility that Patient 7 was the true index case cannot be completely excluded.

The use of real-time WGS could have supported infection control teams, by identifying transmission routes more rapidly and potentially curtailing the spread of the outbreak more effectively.

## Conclusion

This study reports the first outbreaks of OXA-23/NDM-CRAB in metropolitan France, highlighting the circulation of these strains and their ability to cause outbreaks not only in ICUs but also in medical wards. Two clones were involved, all extremely resistant to both first-line and last resort antibiotics, leading to therapeutic challenges. Theses strains are mostly imported from abroad, underscoring the importance of raising awareness among medical staff about screening for MDRO, including CRAB, in patients returning from abroad and admitted to ICUs or medical wards.

## Supplementary Information

Supplementary Material 1

## Data Availability

The dataset supporting the conclusions of this article is available in the NCBI database, under accession number: Bioproject PRJNA1208420 ( https://www.ncbi.nlm.nih.gov/bioproject/1208420).
