# Supplementary material for: Dissemination of OXA-23/NDM co-producing Acinetobacter baumannii in northern Paris hospitals: inter-hospital transmission and screening gaps
Source: Antimicrob Resist Infect Control. 2026 Jan 31;15:32. doi: 10.1186/s13756-025-01694-4 (PMC12947424; doi:10.1186/s13756-025-01694-4)
Supplement: Supplementary file 1 — Supplementary Material 1 [file 13756_2025_1694_MOESM1_ESM.docx]

**Supplemental materials**

**Methods**

*Whole genome sequencing*

Illumina sequencing: Reads quality was checked using FastQC v0.11.8 then reads were trimmed with Trim Galore v0.4.4 (quality > 30, length > 50bp). Assembling the reads was performed using SPAdes v3.12 and assessing hybridization quality by QUAST. *Acinetobacter baumannii* species was confirmed using Metaphlan v4.0.

Nanopore sequencing: Libraries were prepared using SQK-LSK109, EXP-NBD104, EXP-NBD114 kits (Oxford Nanopore Technologies, Oxford, UK).

**Results**

**Figure S1:** Map of Paris showing the geographical location of the five hospitals


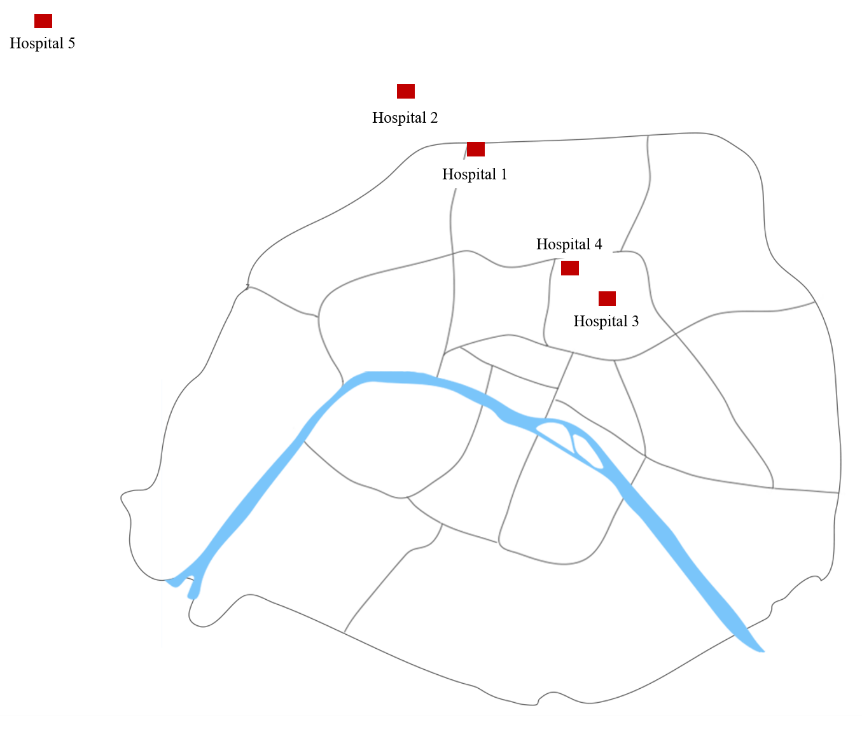


**Figure S2:** SNPs matrix representing the genetic distances between strains belonging to the ST^Ox^1632/^Pas^600.

**Figure S3:** SNPs matrix representing the genetic distances between strains belonging to the ST^Ox^231/^Pas^1. P2_H3 and P4_H3 are considered as not epidemic due to the large SNPs difference with the other strains.

**Figure S2:** Phylogenetic tree based on the core genome of 37 OXA-23/NDM-1-producing *Acinetobacter baumannii* isolated from five adult hospitals in northern Paris between January 2020 and December 2022 and the JACOR strain causing an outbreak on Réunion Island[1]. The tree is unrooted. Strip one represents the patient identification (ID); strips two and three the hospital and ward where the patient was hospitalised when the CRAB was detected, respectively; strip four the country of hospitalisation abroad during the last 3 months; strips five the imported status of the case as confirmed (solid red) or probable (hatched red) ; strip six and seven the ST according to the Oxford and Pasteur schemes, respectively. The two blocks of coloured squares indicate the presence of ARG (orange squares) and virulence genes (purple squares).

**
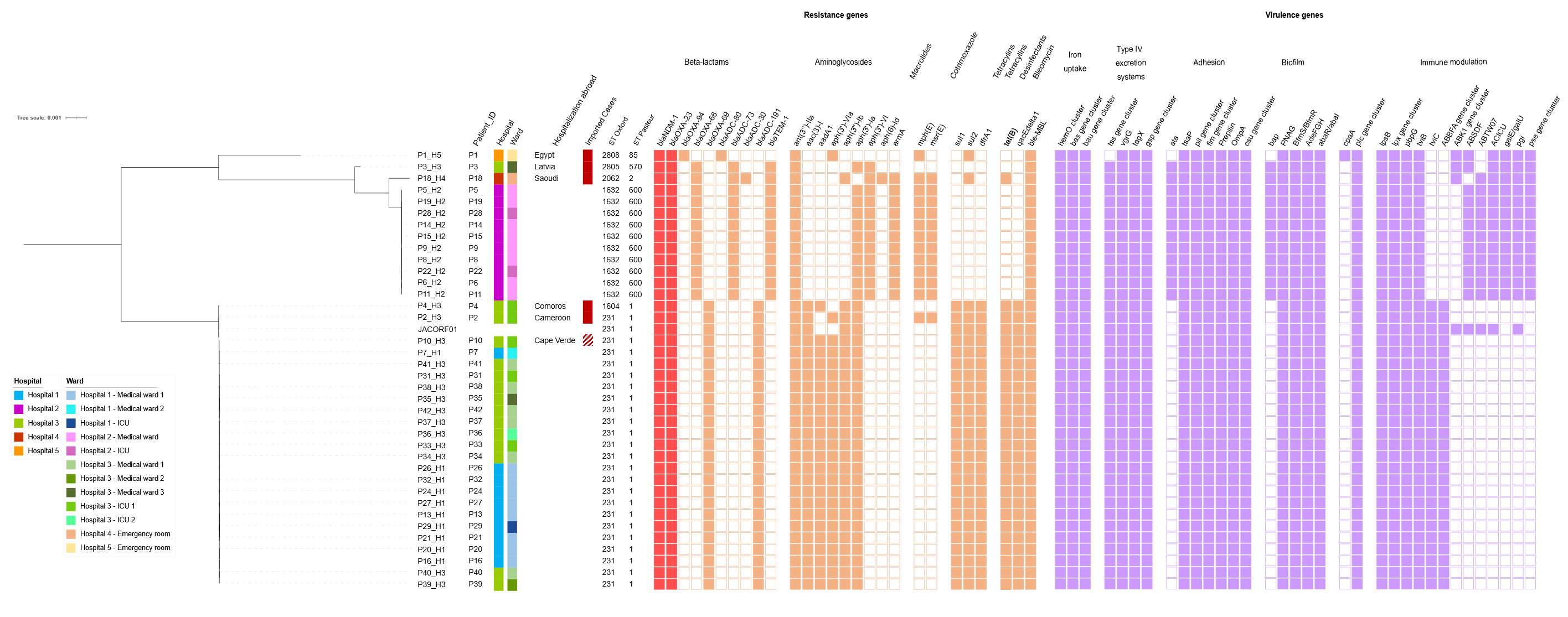
**

1. Miltgen G, Bour M, Allyn J, Allou N, Vedani T, Vuillemenot JB, et al. Molecular and epidemiological investigation of a colistin-resistant OXA-23-/NDM-1-producing Acinetobacter baumannii outbreak in the Southwest Indian Ocean Area. Int J Antimicrob Agents. 2021 Oct;58(4):106402.
